# Go or no-go for treat-to-target in axial spondyloarthritis?

**DOI:** 10.1097/BOR.0000000000000941

**Published:** 2023-04-25

**Authors:** Casper Webers, Marin Been, Astrid van Tubergen

**Affiliations:** a Division of Rheumatology, Department of Internal Medicine, Maastricht University Medical Center; b Care and Public Health Research Institute (CAPHRI), Maastricht University, Maastricht, The Netherlands

**Keywords:** ankylosing spondylitis, axial spondyloarthritis, disease activity, outcome, treat-to-target

## Abstract

For almost a decade, treat-to-target (T2T) has been advocated as a management strategy for axial spondyloarthritis (axSpA), despite a lack of trial evidence. Recently, the first and only published T2T trial in axSpA did not meet its primary endpoint. The purpose of this review is to discuss whether we should continue with a T2T approach in axSpA and to describe some experiences with T2T in clinical practice.

**Recent findings:**

The trial showed no superiority of T2T compared with usual care; however, several secondary trial outcomes and the health economic analysis actually favoured T2T, and there are conceivable reasons for the negative trial results. Furthermore, several knowledge gaps related to an optimal T2T approach in axSpA were identified. In clinical practice, a T2T approach was applied to only a limited extent, possibly because of several challenges.

**Summary:**

Despite one negative trial, it is too early to abandon T2T in axSpA. Not only more evidence from clinical trials but also research on the optimal target and management of all facets of axSpA, are highly needed. For successful implementation of T2T in clinical practice, it is important that barriers and facilitators to application are identified and subsequently addressed.

## INTRODUCTION

For almost a decade, treat-to-target (T2T) has been advocated as a management strategy for axial spondyloarthritis (axSpA) [1,2]. A T2T approach includes several elements such as choosing a target, regular monitoring of this target with validated outcome measures, adapting therapy when the target is not achieved, and shared decision-making. The formulation of the T2T recommendations in axSpA was based on expert opinion and justified by several observational studies. One study demonstrated a longitudinal association between disease activity and progression of radiographic damage in ankylosing spondylitis [3]. Another study showed that the effect of lowering disease activity by tumour necrosis factor (TNF) inhibitors was associated with a decrease in spinal radiographic progression [4]. Achieving inactive disease was associated with almost no radiographic progression in the following 2-year interval and also with improved physical activities and work productivity [4]. However, in contrast to rheumatoid arthritis (RA) and psoriatic arthritis (PsA), in which T2T management strategies have been demonstrated in several randomized controlled trials (RCTs) to be effective for improving outcomes, evidence for T2T from RCTs in axSpA was lacking [5,6]. This argument, in combination with the costs of a T2T strategy, and the burden for patients and rheumatologists, was why the 2019 ACR-SAA-SPARTAN treatment guidelines conditionally recommended against use of a T2T strategy in axSpA [7].

**FIGURE 1:**
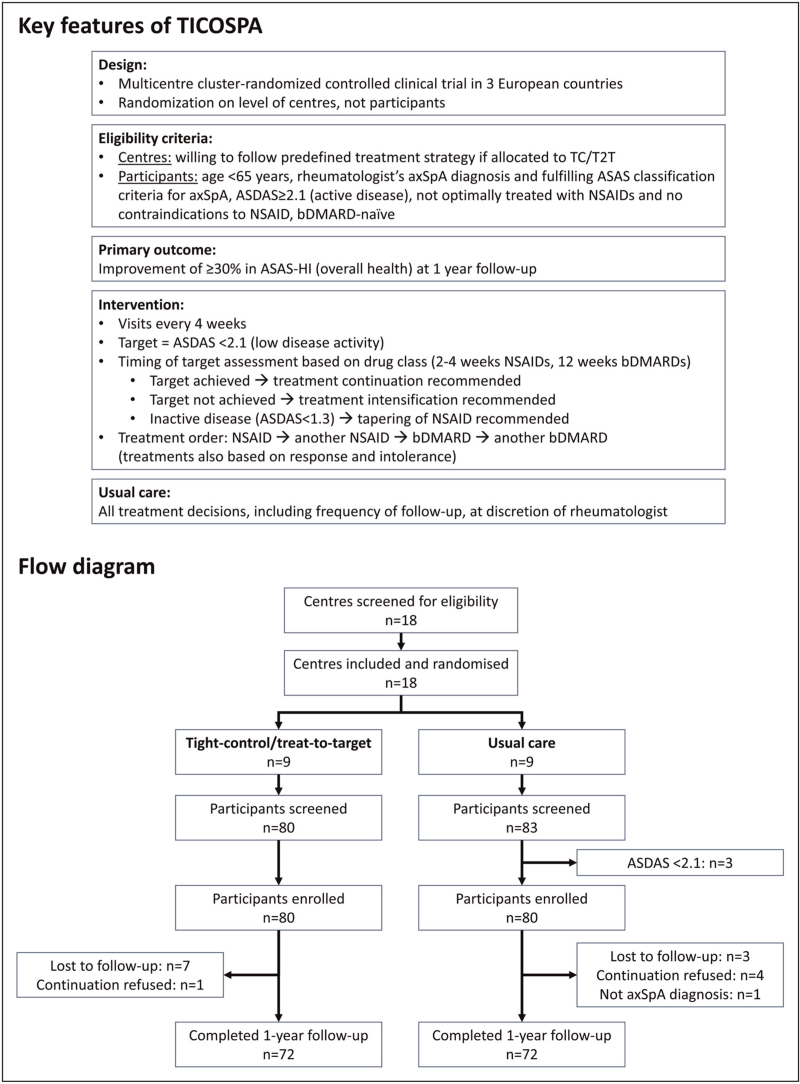
Overview of the key design features (top half) and flow diagram (bottom half) of the Tight Control in SPondyloArthritis (TICOSPA) study. ASAS, Assessment of SpondyloArthritis International Society; ASDAS, Ankylosing Spondylitis Disease Activity Score; axSpA, axial spondyloarthritis; bDMARD, biological disease modifying anti-rheumatic drug; NSAIDs, nonsteroidal anti-inflammatory drugs.

Recently, the results of the first RCT on T2T in axSpA, the Tight Control in SPondyloArthritis (TICOSPA) study, have been published, evaluating the effect of application of tight control/T2T (TC/T2T) towards predefined disease activity targets on health status, compared to routine care (see Fig. 1) [8^▪^]. The primary endpoint, superiority of TC/T2T compared with usual care in the proportion of patients experiencing 30% improvement in the overall functioning and health, was not met. The question is now, should we stop or continue with a T2T approach in axSpA? And if we decide to continue, how should we implement this in clinical practice? In this review, we will discuss arguments for a go or a no-go of T2T in axSpA and describe experiences with application of a T2T approach in axSpA in clinical practice. 

**Box 1:**
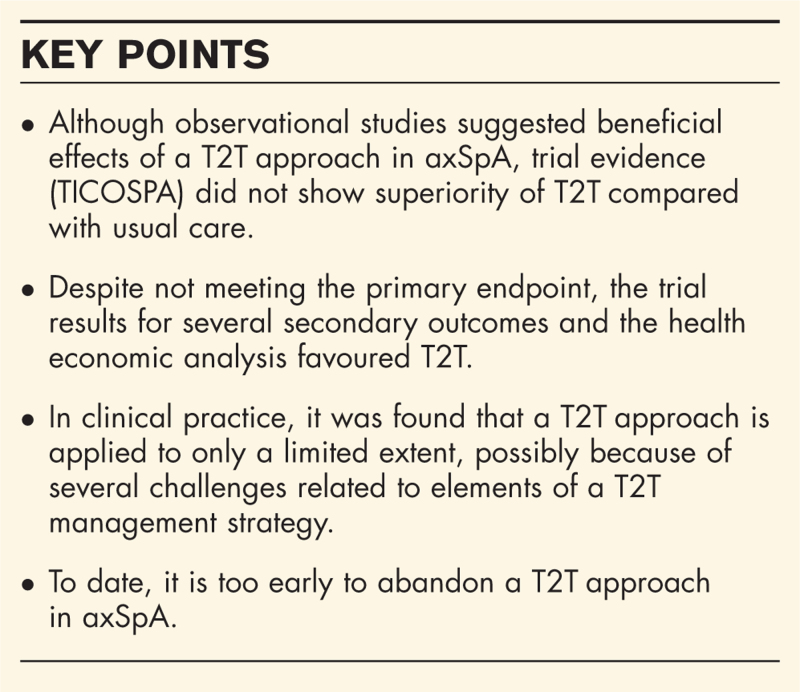
no caption available

## A NO-GO FOR TREAT-TO TARGET IN AXIAL SPONDYLOARTHRITIS

The main argument against a T2T approach in axSpA is the negative result from TICOSPA. Although we had some evidence in favour of a T2T approach from observational studies a decade ago, trial evidence now does not support this. Furthermore, there remain several knowledge gaps related to an optimal T2T approach in axSpA (e.g. what should be the target, how to measure this, what should be the frequency of measurement, when to adapt treatment and which treatment to choose, see Table 1).

**Table 1:** Knowledge gaps related to treat-to-target in axial spondyloarthritis

Choice of target and instrument
Optimal target for T2T strategies in axSpA (ASDAS inactive disease/low disease activity, alternative targets covering extra-musculoskeletal manifestations, imaging)
Suitability of currently endorsed ASDAS cut-offs for T2T strategies in clinical practice
Optimal frequency of monitoring in T2T
Method of disease monitoring in T2T strategies, support tools
Role of remote monitoring (telehealth) in T2T
Treatment
Personalized treatment strategies to achieve target, taking into account disease phenotype and personal context
Effectiveness of T2T strategy in relation to symptom duration
Potential adverse effects of T2T in axSpA (overtreatment, costs)
Effectiveness of a dual target strategy
Tapering/discontinuation of treatment once target has been achieved
Other
Facilitators and barriers to implementation of T2T in clinical practice
Patient perspective/views on T2T strategies in axSpA

ASDAS, Ankylosing Spondylitis Disease Activity Score; axSpA, axial spondyloarthritis; T2T, treat-to-target.

The first steps of a T2T approach are choosing a target and measuring this. Traditionally, the target is remission (or alternatively low disease activity, LDA) and frequent measurement of disease activity is advised. In the international T2T recommendations for axSpA, the target is defined as clinical remission/inactive disease (ID) of musculoskeletal (arthritis, dactylitis, enthesitis, axial disease) and extramusculoskeletal manifestations ([EMMs], inflammatory bowel disease, psoriasis, anterior uveitis) [2]. For axSpA, the preferred instrument to measure disease activity is the AS Disease Activity Score (ASDAS) [2]. The ASDAS combines four patient-reported items related to back pain, peripheral symptoms, morning stiffness, and patient global, with laboratory markers of inflammation (CRP/ESR) [9,10]. Although several key components of axSpA are included, the ASDAS does not contain all manifestations of axSpA. Importantly, EMMs are not covered. These EMMs frequently occur in patients with axSpA, contribute to the total burden of disease and influence treatment decisions [11]. Situations such as inactive disease according to ASDAS, while experiencing a flare of an EMM, can happen in the same individual and, therefore, clinicians cannot solely rely on the ASDAS for making treatment decisions. Furthermore, it is questionable whether the formulated ASDAS cut-off values for high disease activity (HDA, ≥2.1) adequately reflect that a patient is truly in such a disease activity state. A universal and widely accepted ‘gold standard’ for clinical disease activity in axSpA is lacking. In clinical practice, ASDAS cut-offs corresponding to treatment intensification have been shown to be higher (between 2.4 and 3.3) than the formulated HDA cut-off, suggesting that a cut-off of 2.1 might be too strict for clinical practice [12]. Maintaining this target for a T2T approach could potentially lead to overtreatment and may do more harm than good for the individual [13]. Another issue is that in those patients with axSpA who achieve an LDA/ID state according to ASDAS, up to 80% experience clinically relevant symptoms or limitations in functioning and health (Webers *et al.*, unpublished, submitted manuscript, [14]). This phenomenon of so-called ‘residual disease’ suggests that simply aiming to reduce disease activity below a certain target does not guarantee the patient to be in a favourable health state. In particular, the observation that health-related quality of life (HRQoL) is still substantially reduced in many patients with a low ADSAS challenges the potential of a T2T strategy (especially when considering that the goal of treating axSpA is to maximize HRQoL) [15^▪^]. As an alternative, instead of using the absolute value of the ASDAS for a T2T approach, a change score of the ASDAS could be more valuable. For example, a change of 1.1 units is considered as clinically meaningful improvement, and is used as criterion for treatment continuation in the latest ASAS-EULAR recommendations for axSpA [10,15^▪^]. Moreover, a strategy involving two targets (control of inflammation and control of disease impact), as opposed to focusing on disease activity only, might be preferred, although evidence in axSpA is lacking [16].

Another element of T2T is to adapt treatment when the target is not met. Treatment of axSpA is complex because of the heterogeneity of the disease. The recently updated ASAS-EULAR management recommendations underline this challenge and the need for a personalized approach to care [15^▪^]. Although they provide some guidance regarding the treatment choice for specific scenarios – for example, the preferred bDMARD in the presence of specific EMMs – many uncertainties remain about the optimal management strategy in axSpA. Examples include the effectiveness of switching drug classes, the impact of comorbidities on treatment success or how to best manage peripheral manifestations. The currently available treatments for axSpA, although rapidly expanding in number and type over the last decades, on their own do not provide a one-size-fits-all solution.

## A GO FOR TREAT-TO-TARGET IN AXIAL SPONDYLOARTHRITIS

Despite several arguments for a no-go, there are also conceivable counter arguments as to why we should not abandon the T2T principle in axSpA. The results of TICOSPA, the first (and to date, only published) RCT investigating a T2T approach in axSpA, were highly awaited. Previous observational research had provided indirect evidence of the possible benefits of T2T in axSpA, and RCTs in RA and PsA had been successful [3–6]. A high-quality trial, conducted by experts in the field and not hindered by the inherent risk of confounding and bias associated with observational studies, was about to give the final answer on T2T. Perhaps somewhat unexpectedly, the trial failed to meet its primary endpoint, a 30% improvement in overall functioning and health, assessed with the ASAS Health Index (ASAS HI) [8^▪^,^17^]. However, this does not necessarily close the book on T2T in axSpA. In TICOSPA, the target was LDA, while the outcome was functioning and health (a consequence of disease activity). The choice of this outcome was to avoid potential circularity – as opposed to similar trials in RA and PsA, which had used disease activity-related measures as both target and outcome [5,6]. Nonetheless, although the instrument used to assess the outcome (ASAS HI) has been specifically developed to capture the impact of axSpA and has been extensively validated in axSpA, the way it was operationalized in TICOSPA (30% improvement in ASAS HI) was new [18]. This made sample size calculation difficult, possibly resulting in an underpowered trial [18,19]. Indeed, the primary outcome did not significantly differ between groups but was numerically higher in the T2T group (47% TC/T2T versus 36% usual care). The choice of primary outcome might also have contributed to the underestimation of the response rate in the usual care group (25% expected versus 36% observed), although this might also be attributed to potential contamination. As only axSpA expert centres were involved in the trial, it is possible that rheumatologists in the centres randomised to usual care (consciously or subconsciously) delivered care with some elements of a T2T strategy [19,20].

Overall, the choice of the primary outcome in TICOSPA, although well considered, might not have been ideal. Most secondary outcomes, including treatment response measures (ASAS20/40) and measures of physical function and HRQoL, were numerically better in the TC/T2T arm, and for some of these, the between-group difference was also statistically significant [8^▪^]. The latter implies that if one of these outcomes had been chosen as the primary outcome, the trial would have been considered ‘positive’. Another important observation was that the health economic analysis demonstrated that the TC/T2T strategy was favourable from a societal perspective [8^▪^]. Although healthcare costs were somewhat higher in the TC/T2T arm (mainly because of increased bDMARD use), overall costs were actually lower (because of reduced sick leave). This is an important finding, as it negates the argument that TC/T2T will lead to substantially increased costs. One important consideration here is that costs are heavily dependent on regional differences. For TICOSPA, costs were based on the situation in the Netherlands. Both the total costs, and who bears them, will vary. In certain healthcare systems, for example, those involving co-payments, a T2T approach could be associated with increased financial costs for the patient.

Furthermore, it makes sense to target disease activity in the management of axSpA. Observational research has consistently demonstrated the link between disease activity and structural damage to the sacroiliac joints and spine in axSpA and both disease activity and structural damage affect HRQoL [3,4,21]. The nature of disease progression in axSpA, a relatively slow process, might also contribute to the difficulty in showing the benefits of T2T in this disease. If the ultimate goal of T2T is to maximize HRQoL through tight control of disease activity and prevention of structural damage, demonstration of such an effect requires long-term follow-up (i.e. years), whereas trials typically are of shorter duration. This might also (partially) explain why TC/T2T in TICOSPA did not show the expected benefit compared with usual care, as follow-up lasted 48 weeks. It could very well be that T2T in axSpA takes a longer period to show its true benefit.

One argument often posed against T2T in axSpA, is that a substantial proportion of patients – possibly even the majority – will not be able to reach the proposed targets (ASDAS-based ID/LDA). However, over the last two decades, pharmacological treatments for axSpA have expanded rapidly in both number and type [15^▪^]. New drugs targeting varying pathways are also in the development pipeline. Altogether, this suggests that achieving an ID/LDA state will become a reality for an increasing number of patients in the future, making a T2T strategy in axSpA achievable. The main challenge will be to predict which patient requires which type of treatment to achieve their target as quickly as possible.

## IMPLEMENTATION OF TREAT-TO-TARGET IN CLINICAL PRACTICE

Suppose we would go for T2T in axSpA in clinical practice, implementation of this will undoubtedly be challenging. In RA, we have seen that although rheumatologists embrace the T2T principle, actual application into practice is difficult [22,23]. One important barrier for implementation of T2T in practice is the frequency of measuring the target. The international T2T recommendations for SpA advise to regularly measure disease activity, with a frequency depending on prior disease activity scores [2]. In patients who have not achieved the target, re-evaluation of disease activity should take place within 3 months, whereas an interval of 6–12 months may be considered in patients whose target is met. In TICOSPA, patients in the TC/T2T arm were evaluated every 4 weeks. This intensive strategy is very resource demanding. Another barrier may be the measurement of the target itself. Presenting the questionnaire to patients and calculating scores may also be time consuming. Electronic data collection could be a solution to this, although it is questionable whether this really improves application of a T2T approach in clinical practice.

In a study in patients with axSpA, Beckers *et al.* evaluated the extent to which T2T recommendations (i.e. frequency of measurement, target-based treatment intensification) were applied in clinical practice in a setting where care providers were supported by SpA-Net, a web-based patient registry for monitoring SpA in daily practice in the Netherlands [24^▪^]. During a 1-year study period, disease activity was assessed at least once with the ASDAS in 185 out of 219 patients (84%). The frequency of measurement varied from 0 (34 patients) to 6 (1 patient), while the majority (158 patients, 73%) had 1 or 2 measurements during the 1-year follow-up. At the first measurement, 114 (62%) did not meet LDA. Interestingly, in only 26 (23%) of these patients, disease activity was re-evaluated within the recommended 3 months and after 12 months, still in 31 (27%) of the patients, disease activity was not re-evaluated. The researchers also investigated whether treatment adaptation occurred based on the ASDAS state. In 19 out of 114 (17%) patients with HDA, treatment was changed within 6 weeks. At re-evaluation after 3 months in those with persistent HDA, only two more treatment adaptations occurred. From this study, we can conclude that, even with access to a web-based tool for monitoring patients and supporting care providers, T2T is applied to only a limited extent in daily practice in patients with axSpA. The scores seemed not to be driving re-evaluation nor treatment adaptation.

The reasons for this were not investigated in this study, but there are many barriers to consider, which may limit implementation of T2T in clinical practice. For example, there may have been limited time and staff available to comply with frequent disease monitoring. Physicians may have had doubts about the interpretation of the disease activity scores, that is, they experienced uncertainty whether the scores truly reflected disease activity or whether this is attributable to comorbid disease. Finally, patients may have been reluctant to adapt their current treatment, because of worries about potential ineffectiveness or adverse effects of alternative treatment options. For successful implementation of T2T in practice, it is important that such barriers, but also facilitators, to application are identified and subsequently addressed.

## CONCLUSION

One RCT (TICOSPA) failed to show superiority of T2T compared with usual care in axSpA; however, several secondary outcomes and the health economic analysis actually favoured T2T, and there are conceivable reasons why the primary trial endpoint was not met. Moreover, there remain several knowledge gaps related to an optimal T2T approach in axSpA. In clinical practice, a study demonstrated that a T2T approach was applied to only a limited extent, possibly because of several challenges, which need to be identified before T2T can be successfully implemented.

Therefore, we consider it too early to abandon a T2T approach in axSpA. More evidence from clinical trials, but also research on the optimal target and management of all facets of axSpA are highly needed.

## Acknowledgements

*None.*

*This manuscript has been drafted, reviewed, and approved by all contributing authors.*

### Financial support and sponsorship

*A.v.T. has received unrestricted research grants from Novartis, Pfizer, and UCB.*

### Conflicts of interest

*A.v.T. has received unrestricted research grants from Novartis, Pfizer, and UCB, and received consulting fees from Galapagos and Novartis. There are no conflicts of interest for the remaining authors.*

## REFERENCES AND RECOMMENDED READING

Papers of particular interest, published within the annual period of review, have been highlighted as:

▪ of special interest
▪▪ of outstanding interest
